# Effectiveness of Minimal Contact Interventions: An RCT

**DOI:** 10.1016/j.amepre.2020.10.010

**Published:** 2021-03

**Authors:** Samantha Hajna, Stephen J. Sharp, Andrew J.M. Cooper, Kate M. Williams, Esther M.F. van Sluijs, Soren Brage, Simon J. Griffin, Stephen Sutton

**Affiliations:** 1 MRC Epidemiology Unit, Institute of Metabolic Science, University of Cambridge School of Clinical Medicine, Cambridge, United Kingdom; 2 Primary Care Unit, Department of Public Health and Primary Care, University of Cambridge School of Clinical Medicine, Cambridge, United Kingdom

## Abstract

**Introduction:**

Around 23% of adults worldwide are insufficiently active. Wearable devices paired with virtual coaching software could increase physical activity. The effectiveness of 3 minimal contact interventions (paper-based physical activity diaries, activity trackers, and activity trackers coupled with virtual coaching) in increasing physical activity energy expenditure and cardiorespiratory fitness were compared over 12 weeks among inactive adults.

**Methods:**

This was an open label, parallel-group RCT. Inactive adults (aged ≥18 years, N=488) were randomized to no intervention (Control; *n*=121), paper-based diary (Diary; *n*=124), activity tracker (Activity Band; *n*=122), or activity tracker plus virtual coaching (Activity Band PLUS; *n*=121) groups. Coprimary outcomes included 12-week changes in physical activity energy expenditure and fitness (May 2012–January 2014). Analyses were conducted in 2019–2020.

**Results:**

There were no differences between groups overall (physical activity energy expenditure: *p*=0.114, fitness: *p*=0.417). However, there was a greater increase in physical activity energy expenditure (4.21 kJ/kg/day, 95% CI=0.42, 8.00) in the Activity Band PLUS group than in the Diary group. There were also greater decreases in BMI and body fat percentage in the Activity Band PLUS group than in the Control group (BMI= −0.24 kg/m^2^, 95% CI= −0.45, −0.03; body fat= −0.48%, 95% CI= −0.88, −0.08) and in theActivity Band PLUS group than in the Diary group (BMI= −0.30 kg/m^2^, 95% CI= −0.50, −0.09; body fat= −0.57%, 95% CI= −0.97, −0.17).

**Conclusions:**

Coupling activity trackers with virtual coaching may facilitate increases in physical activity energy expenditure compared with a traditional paper‒based physical activity diary intervention and improve some secondary outcomes compared with a traditional paper‒based physical activity diary intervention or no intervention.

**Trial registration:**

This study is registered at www.clinicaltrials.gov ISRCTN31844443.

## INTRODUCTION

It is estimated that 23% of adults worldwide are insufficiently active and at risk of developing inactivity-related chronic diseases.^1^, ^2^, ^3^ Global efforts to increase physical activity (PA) have achieved limited success.^4^ High rates of inactivity are attributed to urbanization and technologic advances that reduce the necessity for activity.^4^, ^5^, ^6^, ^7^ Effective activity promotion strategies will need to leverage these changing environmental and technologic landscapes.

Wearable devices are rapidly advancing technologies that could facilitate increases in PA.^8^, ^9^, ^10^ Worldwide, 71 million smartwatches were sold in 2018.^11^ This number is expected to reach 140 million by 2022.^11^ Activity tracking interventions provide individualized feedback and are easier to implement and more scalable than traditional behavioral interventions (e.g., support groups).^12^ There is some evidence that trackers may help adults increase PA^13^, ^14^, ^15^, ^16^ and lose body mass.^17^^,^^18^ It remains unclear whether trackers used in conjunction with virtual coaching are better than trackers used alone and how these compare with more traditional interventions. It also remains unclear whether these benefits extend to objective measures of PA energy expenditure (PAEE) and cardiorespiratory fitness, both of which have been linked to a decreased risk of premature mortality.^19^, ^20^, ^21^ Identifying effective, scalable, and cost-effective strategies for increasing PAEE and fitness would strengthen the rationale for their use in clinical and public health settings.

The primary aim of this study is to evaluate the effectiveness of 3 minimal contact interventions (paper-based PA diary, activity tracker, and activity tracker coupled with a user-driven online virtual coaching platform) on objectively assessed PAEE and cardiorespiratory fitness. The secondary aim is to assess the effect of these interventions on clinical markers of cardiometabolic health, self-reported health measures, and psychological measures related to PA self-monitoring.

## METHODS

Get Moving is an open-label, parallel-group RCT (ISRCTN31844443). Ethical approval was granted by the Cambridge Central National Health Service Research Ethics Committee (Reference 09/H0308/3). The trial is presented in accordance with the CONSORT statement.

### Study Population

As reported elsewhere,^22^ a generic advertisement was circulated through newsletter, e-mail, posters, and intranet to staff and students on the Cambridge Biomedical Campus (Cambridge, United Kingdom). The advertisement targeted staff/students who wished to become more active or were interested in learning how fit they were. Recruitment stands were also run in areas frequented by staff/students (e.g., canteens). Interested individuals completed a brief questionnaire. Exclusion criteria included a score ≥30 on the Godin Leisure-Time Exercise Questionnaire for moderate- to vigorous-intensity PA,^23^ being aged <18 or >65 years, being advised by a medical professional not to engage in regular PA, inability to walk for ≥15 minutes unaided, participation in another trial, taking ≥100 mg/day of a β-blocker, pregnancy, leaving the campus in ≤4 months, and inability to use a computer or English-language website. Eligible individuals were invited to attend a baseline assessment where they provided written informed consent and were further excluded if they had a BMI ≤18 kg/m^2^ or blood pressure ≥160/100 mmHg.

Follow-up of 100 participants per group enabled the detection of differences of 0.025 kJ/kg/minute (36 kJ/kg/day) and 0.19 L/minute (1.7 mL oxygen/kg/minute) with 80% power at a 5% significance level. The aim was to recruit 480 participants, with 120 participants in each of the 4 groups at baseline to allow for an attrition rate of up to 17% on the basis of previous studies.^24^^,^^25^

After the baseline assessment, participants wore a combined uniaxial accelerometer–heart rate monitor (Actiheart) for 6 consecutive days and nights. Participants returned their monitors by prepaid post or by delivering them to the study center. Data were checked for quality and quantity (≥35 hours of wear). Monitors were worn again if they were returned with insufficient data. Participants were withdrawn from the study if they returned their monitor a second time with insufficient data.

Participants were randomized (March 01, 2012–October 11, 2013) into intervention groups using randomization lists prepared in Stata within strata defined by age (<45 and ≥45 years), sex, and BMI (<27 and ≥27 kg/m^2^) and a block size of 8. The Control and Diary groups were sent their allocation by mail. Research assistants told the Activity Band and Activity Band PLUS group participants their group allocations, gave them their study equipment, and gave them specific instructions on what to do during the intervention period at their places of work/study.

Participants were contacted by telephone at 8 weeks after randomization. They were asked to wear the Actiheart at Week 10 for another 6 days and to return it by prepaid post or by delivering it to the study center immediately after the 6-day monitoring period. They were also asked to attend a 12-week follow-up assessment (May 21, 2012–January 31, 2014). The measurement team was unaware of group allocation. Participants were masked to all clinical measures.

### Interventions

The Control group did not have an intervention. In the Diary group, participants received a pocket-sized paper PA diary to record the daily activity of ≥10 minutes (yes/no), the activity type, the time the activity began, and the activity duration for 12 weeks.

Participants in the Activity Band group received a triaxial wrist-worn activity tracker that contained an accelerometer similar to one described previously.^26^ The activity tracker was developed by Imperative Health, which was a part of AXA ICAS Limited, an employee assistance program, health, and well-being services provider.^27^ The tracker could store data for up to 2 weeks. Participants were instructed to start wearing their tracker immediately, activate their online Imperative Health account within 7 days, upload their activity data by Bluetooth, and view their activity graphs ≥1 per week. Graphs showed the duration (minutes) and intensity (moderate, high, very high) of PA undertaken (daily, weekly, monthly). The movement intensity cut offs were based on absolute acceleration vector magnitude, were expressed in arbitrary units, and were universal across age and sex groups.

Participants in the Activity Band PLUS group received the same intervention as those in the Activity Band group, in addition to Bluetooth-enabled scales to self-monitor body weight, a healthy meals recipe book, access to Imperative Health's online nutritional program for monitoring calorie intake, and access to Imperative Health's web-based automated dialogue system, which served as a virtual coach. The virtual coach was a user-driven online platform and provided the Activity Band PLUS group a more interactive experience than that provided to the Diary and Activity Band groups. It used the activity data from the activity trackers and the weight data from the Bluetooth-enabled scales to provide numerical and graphical feedback to participants regarding their progress. The system also contained structured daily meal plans, a food diary, a PA planner, progress graphs with PA levels and weight loss goals displayed in graphical format, and a tailored message service that provided reminders for exercise and feedback to encourage the adoption and maintenance of PA through the setting and meeting of goals. Tailored messages were delivered to participants through Imperative Health's online platform and were based on information entered into the system by the participants. As with the Activity Band group, participants were instructed to activate their account within 7 days. They were also instructed to upload their movement data to their accounts and to view their activity graphs ≥1 per week. No specific instructions were given on whether they were to monitor their weight and caloric intake or whether to use the recipe book. Neither did this group have any specific weight loss goals. Participants were only told to use each component of the intervention as much as they wished.

For the Activity Band and the Activity Band PLUS groups, if no account activity was detected for 15 consecutive days, Imperative Health informed the study team, who contacted the participant to find whether they were experiencing technical difficulties or were no longer interested in participating in the study.

### Measures

With the exception of the strength of habit for PA, conscientiousness, and psychologically becoming more physically active variables that were assessed using existing tools,^28^, ^29^, ^30^ descriptive characteristics were assessed using a questionnaire developed for this study (Table 1). Participants were instructed to abstain from nicotine, caffeine, and vigorous exercise for 1 hour before the baseline and follow-up visits. Coprimary outcomes were assessed at both visits. Secondary outcomes were assessed at both visits with the exception of the leisure-time PA and PA self-monitoring psychological measures (assessed at 12-week follow-up and 2 weeks after randomization, respectively). The study timeline is provided in Appendix Figure 1 (available online).

**Table 1:** Baseline Characteristics of the Study Population

Characteristics	Control	Diary	Activity Band	Activity Band PLUS
Participants,^a^*n*	121	124	122	121
Age, years, mean (SD)	43.7 (10.4)	44.4 (11.3)	42.0 (10.8)	42.5 (11.1)
BMI, kg/m^2^, mean (SD)	26.9 (5.6)	26.6 (4.9)	27.2 (5.7)	26.4 (4.8)
Strength of habit for physical activity^b^ (range: 1‒5), mean (SD)	2.6 (0.9)	2.5 (0.9)	2.5 (0.8)	2.5 (0.8)
Conscientiousness^c^ (range: 1‒7), mean (SD)	2.1 (1.1)	2.4 (1.1)	2.3 (1.1)	2.5 (1.3)
Women, % (*n*)	84.3 (102)	83.9 (104)	82.8 (101)	83.5 (101)
Current/ever smoker (versus never), % (*n*)	67.0 (59)	60.2 (56)	56.4 (53)	58.3 (49)
White ethnicity (versus other), % (*n*)	88.3 (106)	86.2 (106)	82.6 (100)	88.4 (107)
Degree or higher (versus A level/General Certificate of Secondary Education/no qualification), % (*n*)	59.5 (72)	62.9 (78)	69.4 (84)	68.6 (83)
Living alone status, % (*n*)	8.3 (10)	23.4 (29)	16.7 (20)	12.4 (15)
≥1 children aged <18 years in household, % (*n*)	39.7 (48)	34.7 (43)	32.0 (39)	41.3 (50)
Gym membership, % (*n*)	36.4 (44)	39.5 (49)	44.3 (54)	43.8 (53)
Vitamin C supplement use in the last 7 days, % (*n*)	13.2 (16)	13.8 (17)	12.3 (15)	7.4 (9)
Multivitamin supplement use in the last 7 days, % (*n*)	13.2 (16)	18.5 (23)	19.7 (24)	15.0 (18)
Prescribed medication use, % (*n*)	47.1 (56)	52.8 (65)	50.8 (62)	45.8 (55)
Frequency of self-weighing over the past 12 weeks, more than once per month, % (*n*)	42.1 (51)	42.7 (53)	37.7 (46)	46.3 (56)
Psychological measures related to becoming physically active^d^ (range: 1‒7), mean (SD)				
Attitudes	6.0 (0.8)	5.9 (0.8)	5.9 (0.8)	5.9 (0.7)
Intentions	5.5 (1.3)	5.4 (1.4)	5.5 (1.3)	5.5 (1.4)
Subjective norms	4.8 (1.5)	4.9 (1.4)	4.9 (1.2)	4.7 (1.4)
Perceived behavioral control	6.3 (1.0)	6.2 (1.0)	6.3 (1.0)	6.3 (1.0)

a Number of participants, if sample size varied: strength of habit for physical activity: Diary=123; conscientiousness: Activity Band PLUS=120; current/ever smoker: Control=88; Diary=93; Activity Band=94; Activity Band PLUS=84; ethnicity: Control=120; Diary=123; Activity Band=121; education: Activity Band=121; living alone: Activity Band=120; vitamin C supplement use: Diary=123; multivitamin supplement use: Activity Band PLUS=120; prescribed medication use: Control=119; Diary=123; Activity Band PLUS=120.

b Strength of habit for physical activity was assessed using a 12-item tool that evaluated past behaviors, automaticity, and identity expression using a 5-point scale (1: strongly disagree; 5: strongly agree).^28^ A higher score represented a greater strength of habit for physical activity.

c Conscientiousness was assessed on the basis of ratings of 2 items (I see myself as dependable, self-disciplined; I see myself as disorganized, careless) on a 7-point scale (1: disagree strongly; 7: agree strongly). A lower index was indicative of greater conscientiousness.

d Attitudes, intentions, subjective norms, and perceived behavioral control related to becoming more physically active were assessed using a modified Theory of Planned Behavior Questionnaire.^22^^,^^42^ Final scores were calculated for each construct by averaging the scores of the items related to each of the 4 constructs (Appendix Table 1, available online). Higher scores were indicative of more positive attitudes, intentions, norms, and perceived behavioral control related to becoming more physically active.

Participant PAEE was assessed for 6 consecutive days and nights using Actiheart monitors mounted on 2 chest-mounted electrodes.^31^ Participants were provided with verbal and written instructions on how to use the monitor. After the monitoring period, participants returned the monitors to the study center, or a research assistant collected them at the participant's place of work/study. The heart rate data were preprocessed to remove noise^32^ and individually calibrated to energy expenditure using the heart rate response to a ramped treadmill test.^33^ This was combined with acceleration in a branched equation framework^34^ and used to estimate activity intensity (J/minute/kg). The resulting time-series data were summarized as PAEE (kJ/kg/day).^35^

Fitness was assessed using heart rate response to a submaximal ramped treadmill protocol (T Gonzales, unpublished data, February 2020).^33^ The test ended when participants requested to stop or when 80% of the age-predicted maximum heart rate was reached.^36^ Oxygen consumption per kg body mass was predicted from treadmill speed and incline.^33^ This was regressed on observed heart rate and extrapolated to age-predicted maximum heart rate to provide an estimate of fitness (mL of oxygen/kg/minute). This approach does not include division by individually observed body mass and hence eliminates the potential for bias in the measure for change in fitness resulting from weight change alone.

Body mass and body fat percentage were measured using a bioelectrical impedance monitor (Tanita BC-418MA), and height was measured using a wall-mounted stadiometer. Waist circumference was estimated as the average of 2 measurements taken with a D-loop measuring tape placed halfway between the anterior superior iliac crest and the lowest point of the rib cage. Blood pressure was calculated as the average of 3 measurements assessed after 10 minutes of rest using an automatic sphygmomanometer (Omron), with participants in a seated position and the cuff placed at heart level on the dominant arm as per the MRC Epidemiology Unit's Standard Operating Procedures. BMI was calculated as kg/m^2^ on the basis of the body mass and height measures. All blood markers were assessed using a closed blood collection system (Monovette) (Table 2).

**Table 2:** Intervention Effects on the Secondary Outcomes (Mean Differences, 95% CIs)

Variable	Diary versus Control	Activity Bband versus Control	Activity Band PLUS versus Control	Activity Band versus Diary	Activity Band PLUS versus Diary	Activity Band PLUS versus Activity Band
Differences in mean changes between groups in the secondary outcomes that were assessed at baseline and at 12 weeks follow-up						
BMI, kg/m^2^	0.06 (‒0.15, 0.26)	‒0.05 (‒0.25, 0.16)	‒0.24 (‒0.45, ‒0.03)	‒0.11 (‒0.31, 0.10)	‒0.30 (‒0.50, ‒0.09)	‒0.19 (‒0.40, 0.02)
Body fat, %	0.09 (‒0.30, 0.49)	‒0.08 (‒0.48, 0.32)	‒0.48 (‒0.88, ‒0.08)	‒0.17 (‒0.57, 0.23)	‒0.57 (‒0.97, ‒0.17)	‒0.40 (‒0.80, 0.002)
Waist circumference, cm	‒0.68 (‒3.84, 2.48)	‒2.97 (‒6.16, 0.21)	‒0.74 (‒3.93, 2.45)	‒2.29 (‒5.46, 0.87)	‒0.06 (‒3.23, 3.11)	2.23 (‒0.97, 5.43)
Systolic blood pressure, mmHg	1.10 (‒1.13, 3.33)	0.17 (‒2.07, 2.42)	0.18 (‒2.07, 2.43)	‒0.93 (‒3.16, 1.30)	‒0.92 (‒3.16, 1.32)	0.01 (‒2.25, 2.26)
Diastolic blood pressure, mmHg	0.55 (‒0.90, 1.99)	‒0.33 (‒1.79, 1.13)	‒0.72 (‒2.18, 0.75)	‒0.88 (‒2.32, 0.57)	‒1.26 (‒2.71, 0.19)	‒0.39 (‒1.85, 1.08)
Plasma vitamin C, μmol/L^a^	1.15 (‒3.92, 6.23)	‒1.67 (‒6.75, 3.42)	‒1.14 (‒6.26, 3.98)	‒2.82 (‒7.89, 2.26)	‒2.30 (‒7.41, 2.81)	0.52 (‒4.59, 5.64)
HbA1c, mmol/mol^b^	‒0.19 (‒0.69, 0.32)	0.03 (‒0.47, 0.54)	0.14 (‒0.37, 0.65)	0.22 (‒0.29, 0.72)	0.33 (‒0.18, 0.84)	0.11 (‒0.40, 0.62)
Fructosamine, micromol/L^a^	3.96 (‒2.77, 10.7)	0.10 (‒6.68, 6.88)	1.41 (‒5.37, 8.19)	‒3.86 (‒10.61, 2.89)	‒2.55 (‒9.30, 4.20)	1.31 (‒5.49, 8.11)
Total cholesterol, mmol/L^b^	0.06 (‒0.09, 0.21)	0.01 (‒0.14, 0.16)	‒0.08 (‒0.23, 0.07)	‒0.05 (‒0.20, 0.10)	‒0.14 (‒0.29, 0.01)	‒0.09 (‒0.24, 0.06)
HDL cholesterol, mmol/L^b^	0.03 (‒0.03, 0.09)	0.04 (‒0.02, 0.09)	0.01 (‒0.05, 0.07)	0.01 (‒0.05, 0.06)	‒0.02 (‒0.08, 0.04)	‒0.03 (‒0.08, 0.03)
LDL cholesterol, mmol/L^b^	0.05 (‒0.08, 0.17)	‒0.04 (‒0.17, 0.09)	‒0.04 (‒0.17, 0.08)	‒0.09 (‒0.22, 0.04)	‒0.09 (‒0.22, 0.04)	‒0.004 (‒0.13, 0.13)
Total/HDL cholesterol ratio, %^b^	‒0.06 (‒0.19, 0.08)	‒0.08 (‒0.22, 0.05)	‒0.09 (‒0.22, 0.05)	‒0.03 (‒0.16, 0.11)	‒0.03 (‒0.17, 0.10)	‒0.01 (‒0.14, 0.13)
Triglycerides^c^	0.99 (0.89, 1.10)	1.00 (0.90, 1.11)	0.89 (0.80, 0.99)	1.01 (0.91, 1.12)	0.90 (0.81, 1.00)	0.89 (0.80, 0.99)
Physical functioning^d^	‒1.21 (‒2.98, 0.56)	‒0.03 (‒1.80, 1.75)	‒0.53 (‒2.31, 1.26)	1.19 (‒0.58, 2.95)	0.69 (‒1.09, 2.46)	‒0.50 (‒2.28, 1.28)
Mental functioning^d^	‒0.31 (‒2.48, 1.85)	‒1.53 (‒3.70, 0.65)	1.21 (‒0.97, 3.40)	‒1.21 (‒3.37, 0.95)	1.53 (‒0.64, 3.70)	2.74 (0.56, 4.92)
Perceived stress scale (range: 0‒16)^e^	0.14 (‒0.55, 0.83)	0.25 (‒0.45, 0.94)	‒0.24 (‒0.93, 0.46)	0.11 (‒0.58, 0.79)	‒0.38 (‒1.07, 0.31)	‒0.48 (‒1.18, 0.21)
Mean differences between groups in secondary outcomes that were assessed only once during follow-up						
Leisure-time physical activity, MET hours/day^f^	‒1.31 (‒2.44, ‒0.17)	‒0.60 (‒1.74, 0.54)	‒0.47 (‒1.61, 0.67)	0.71 (‒0.43, 1.84)	0.84 (‒0.30, 1.98)	0.13 (‒1.01, 1.28)
Physical activity self-monitoring (range: 1‒7)^g^						
Attitudes	‒0.15 (‒0.41, 0.11)	0.06 (‒0.20, 0.32)	0.13 (‒0.13, 0.39)	0.20 (‒0.06, 0.47)	0.28 (0.02, 0.54)	0.08 (‒0.19, 0.34)
Intentions	0.30 (‒0.05, 0.64)	0.26 (‒0.09, 0.61)	0.52 (0.18, 0.87)	‒0.04 (‒0.39, 0.31)	0.23 (‒0.12, 0.57)	0.26 (‒0.09, 0.61)
Subjective norms	0.50 (0.13, 0.87)	0.20 (‒0.18, 0.57)	0.36 (‒0.01, 0.73)	‒0.30 (‒0.68, 0.07)	‒0.14 (‒0.51, 0.24)	0.17 (‒0.21, 0.54)
Perceived behavioral control	‒0.17 (‒0.44, 0.09)	‒0.10 (‒0.37, 0.17)	0.06 (‒0.21, 0.32)	0.08 (‒0.19, 0.35)	0.23 (‒0.04, 0.50)	0.15 (‒0.12, 0.42)

a Analyses of plasma vitamin C and fructosamine were conducted by the National Institute for Health Research Biomedical Research Centre's Core Biochemistry Assay Laboratory (Cambridge, United Kingdom).

b Analyses of HbA1c and cholesterol were conducted by the Department of Clinical Biochemistry (University of Cambridge, United Kingdom).

c Intervention effects for triglycerides are presented as ratios of geometric means, and therefore, 1 is the value corresponding to no effect.

d Assessed using the SF-8 Health Survey, which consisted of 8 items that evaluated general health, physical functioning, the effect of physical functioning on daily life/work, bodily pain, vitality, social functioning, emotional health, and the effect of emotional functioning on daily life/work.^37^^,^^38^ Physical and mental functioning scores were calculated by weighting each item using a norm-based scoring method and summing these weighted scores to produce overall physical and mental functioning scores.^37^^,^^74^ Higher scores indicated better physical and mental functioning.

e Assessed using the PSS-4, which consisted of 4 items that assessed perceived stress in the last month (*How often have you felt that you were unable to control the important things in your life*?; *How often have you felt confident about your ability to handle your personal problems*?; *How often have you felt that things were going your way*?; *How often have you felt difficulties were piling up so high that you could not overcome them*?).^39^ Responses were provided on a 5-point scale ranging from *never* to *often*. The responses were summed to produce a final score where a higher score indicated higher perceived stress.

f Assessed using the Recent Physical Activity Questionnaire^40^ at 12 weeks of follow-up. Activities used in the calculation of leisure-time physical activity included swimming, backpacking, walking for pleasure, cycling, gardening, do-it-yourself activities, fitness/exercising activities, dancing, running, bowling, tennis/badminton, squash, table tennis, golf, football/rugby/hockey, cricket, rowing, netball/volleyball/basketball, fishing, horse riding, snooker/billiards/darts, musical instruments playing/singing, ice skating, sailing/windsurfing/boating, and martial arts/boxing/wrestling.

g Attitudes, intentions, subjective norms, and perceived behavioral control related to physical activity self-monitoring were assessed at 2 weeks after randomization using a modified Theory of Planned Behavior Questionnaire.^22^^,^^41^ Final scores were calculated for each construct by averaging the scores of the items related to each of the 4 constructs (Appendix Table 1, available online). Higher scores were indicative of more positive attitudes, intentions, norms, and perceived behavioral control related to physical activity self-monitoring. HDL, high-density lipoprotein; LDL, low-density lipoprotein; PSS-4, Perceived Stress Scale 4; SF-8 Health Survey, Short Form-8 Health Survey.

Physical and mental functioning was assessed using the Short Form-8 Health Survey.^37^^,^^38^ Perceived stress over the last month was assessed using the Perceived Stress Scale 4.^39^ Higher scores were indicative of better physical/mental functioning and higher stress. Leisure-time PA (MET hours per day) over the last 4 weeks was assessed using the Recent Physical Activity Questionnaire.^40^

A modified version of the Theory of Planned Behavior Questionnaire was used to assess attitudes, intentions, subjective norms, and perceived behavioral control related to PA self-monitoring (Appendix Table 1, available online).^22^^,^^41^ Higher scores were indicative of more positive attitudes, intentions, subjective norms, and perceived behavioral control.

Adherence to the Actiheart protocol was based on the number of wear days. Season was based on the activity monitoring dates. Reasons for study participation were queried in the baseline questionnaire. Days off work due to illness, job satisfaction, contact with other participants, self-monitoring behaviors, and the use of PA advice websites were assessed at the 12-week follow-up visit. Adverse events were recorded over the follow-up period.

### Statistical Analysis

Participants were included in the groups to which they were randomized. Descriptive statistics were calculated for all variables, including baseline and follow-up levels of PAEE and fitness, and calculated for corresponding absolute changes over the 12-week follow-up. Differences in mean change between groups in the coprimary outcomes were estimated using a repeated measures ANCOVA model. The model included 3 indicator variables for the randomized group, an indicator variable for time (baseline or 12-week follow-up), 3 randomized group X time interaction parameters, baseline value of the outcome, and a random intercept. The difference in mean change comparing randomized groups was estimated from the interaction parameters. A Wald test was used to test the null hypothesis of no overall difference between randomized groups. Interactions between the randomized group and (1) sex and (2) BMI (below/above median) were tested by including 3-way interaction parameters in the model and performing a Wald test. Secondary outcomes were analyzed using the same model, but no statistical tests were performed. Triglyceride values were log transformed before analysis owing to their skewed distribution, and therefore, differences between groups in these values are reported as ratios of geometric means. Where secondary outcomes were only assessed at follow-up and not baseline, linear regression was used to estimate the differences between groups.

A sensitivity analysis was used to check whether adjusting for randomization stratifiers affected the conclusions of the coprimary outcome analyses. In a secondary analysis based on a per-protocol population, participants were included if they had recorded activity twice per week for ≥4 weeks (Diary) or wore the Activity Band twice per week and logged into the Imperative Health website once per week for ≥4 weeks (Activity Band and Activity Band PLUS). A post hoc analysis was performed for PAEE in which only participants from the per-protocol population who had accrued ≥48 hours of activity–heart rate data with ≥8 hours of data in each quadrant of the day were included. Analyses were performed in 2019–2020 using Stata, version 16.1.

## RESULTS

**Figure 1:**
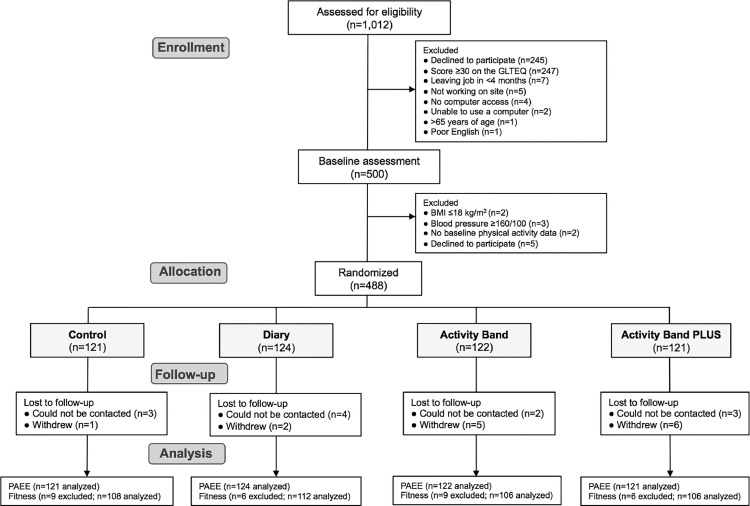
CONSORT flowchart.

*Note*: Participants were only excluded from the analyses if both baseline and follow­up measures were missing. GLTEQ, Godin Leisure-Time Exercise Questionnaire; PAEE, physical activity energy expenditure.

Of the 500 individuals who attended the baseline assessment, 488 were randomized (Figure 1; the numbers of individuals with follow-up data are provided in Appendix Table 2, available online). With only a few exceptions, baseline characteristics were similar across groups (Table 1).

**Figure 2:**
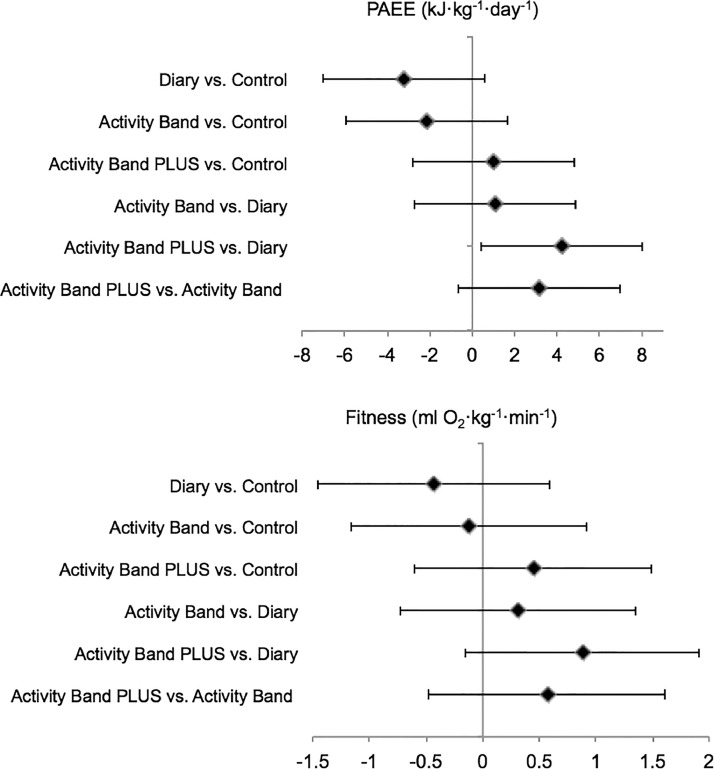
Intervention effects on PAEE and cardiorespiratory fitness.

*Note*: Values represent baseline-adjusted differences in mean changes among groups (95% CIs). Sample sizes: PAEE model=488; fitness model=432. PAEE, physical activity energy expenditure.

With the exception of the Diary group, PAEE and fitness increased in all groups. The largest increases were observed in the Activity Band PLUS group (Appendix Figure 2, available online). Intervention effects are shown in Figure 2 with corresponding effects and CIs provided in Appendix Table 3 (available online). There was no overall evidence of differences between groups (PAEE: *p*=0.114, fitness: *p*=0.417). There was, however, a greater increase in PAEE in the Activity Band PLUS than in the Diary group (PAEE: 4.21 kJ/kg/day, 95% CI=0.42, 8.00). There were no interactions by sex (PAEE: *p*=0.084, fitness: *p*=0.361) or BMI (PAEE: *p*=0.490, fitness: *p*=0.772). Adjusting for the randomization stratifiers made little difference to the results (Appendix Table 3, available online).

The Activity Band PLUS group had greater reductions in BMI, body fat percentage, and triglycerides than the Control group; greater reductions in triglycerides than the Activity Band group; and greater reductions in BMI and body fat percentage than the Diary group. There was no conclusive evidence of benefits in the Diary or Activity Band groups (Table 2).

There were no intervention effects on physical and mental functioning or perceived stress. Leisure-time PA at 12 weeks was lower in the Diary than in the Control group (Table 2).

At 2 weeks after randomization, the Activity Band PLUS group had more favorable intentions than the Control group and more favorable attitudes than the Diary group. The Diary group believed more strongly than the Control group that others thought that it was important that they paid attention to the amount of activity that they would do over the next 12 weeks (Table 2).

The per-protocol results were similar to the main results. No important differences in the PAEE results were observed when retaining participants only if they accrued ≥48 hours of activity–heart rate data with ≥8 hours of data in each quadrant of the day (Appendix Table 4, available online).

At follow-up, days off work, job satisfaction, and contact with other participants did not differ across groups (Appendix Table 5, available online). The Activity Band PLUS group had higher percentages of participants tracking their PA (70.2% vs 47.8%), self-weighing (74.6% vs 45.2%), and using PA advice websites (51.8% vs 22.6%) than the Activity Band group. The Activity Band PLUS group also had higher percentages of participants tracking their PA, self-weighing, and using PA advice websites than the Diary and Control group participants—with the exception of PA tracking, which was higher in the Diary group than in the Activity Band PLUS group (83.1% vs 70.2%). All participants wore the Actiheart for ≥2 days at baseline; 88.9% wore the sensor for ≥2 days at follow-up. Percentages of participants wearing sensors for ≥2 days at follow-up were similar across groups (Control group: 88.4%, Diary group: 89.5%, Activity Band group: 90.2%, Activity Band PLUS group: 87.6%). Seasons of assessments were balanced across groups (Appendix Table 6, available online). Participants joined the study to increase PA (55.4%), to lose weight (29.9%), to maintain weight (3.9%), or for other/unspecified reasons (10.8%). A total of 10 adverse events occurred (Control group=2, Diary group=1, Activity Band group=5, Activity Band PLUS group=2). A total of 4 adverse events were as a result of participants breaking a bone (1 per group). The remainder included rashes from Actiheart electrodes, arthritis, and fainting during a blood draw.

## DISCUSSION

There was no overall evidence of differences among groups for the coprimary outcomes. There were, however, greater increases in PAEE in the Activity Band PLUS group than in the Diary group. In terms of the secondary outcomes, the Activity Band PLUS group had greater reductions in BMI and body fat percentage than the Control group and greater reductions in BMI and body fat percentage than the Diary group. Compared with the Diary group, the Activity Band PLUS group also had more PA self-monitoring‒positive attitudes. Compared with the Control group, the Activity Band PLUS group had stronger intentions to pay attention to how much PA they would do in the next 12 weeks. Compared with the Control group, the Diary group reported accumulating less leisure-time PA at follow-up and believed more strongly that others thought that it was important that they paid attention to the amount of activity that they would do over the next 12 weeks.

This study is the first, to the authors’ knowledge, to examine the effectiveness of activity trackers coupled with virtual coaching in increasing PAEE and fitness compared with the effectiveness of the more traditional interventions. No overall differences in intervention effects were observed. This may have been due to minimal differences in the Theory of Planned Behavior Questionnaire variables—hypothesized mediating variables.^42^, ^43^, ^44^, ^45^ This is consistent with a work that has suggested that there is limited evidence that these variables mediate changes in PA.^46^ Pathways through which activity tracking coupled with virtual coaching might lead to improvements in clinical outcomes should be investigated further. Despite no overall differences, a greater increase in PAEE was identified in the Activity Band PLUS group than in the Diary group (4.21 more kJ/kg/day). In a study of adults followed for a median of 12.5 years, each 10 kJ/kg/day difference in baseline PAEE was associated with a 30% decreased risk for all-cause mortality (hazard ratio=0.70, 95% CI=0.64, 0.78).^47^ Although the difference in PAEE observed in these analyses was slightly smaller (i.e. <10 kJ/kg/day), given that the health benefits of energy expenditure may be graded, this smaller difference may have some benefits.^48^

There were 2 unexpected findings. First, PAEE and fitness increased in the Control group. Although this may have been due to participants modifying their behaviors because of being enrolled in a study, this is unlikely because no increase in PAEE was observed in the Diary group. Other explanations include chance findings or that the participants in the Control group engaged in more health-promoting behaviors to compensate for not having received an intervention. The latter is consistent with the finding that the Control group reported more contact with other study participants and more self-weighing than the Diary group. The second unexpected finding was that the Diary group was the only group not to increase PAEE despite evidence that they believed more strongly than the Control group that others thought that it was important that they paid attention to the amount of activity that they did over the next 12 weeks. This suggests that the Diary intervention was ineffective in increasing PAEE over the follow-up period and is consistent with the finding that the Diary group reported less leisure-time PA at follow-up than the Control group.

Participants in the Activity Band PLUS group had greater reductions in BMI and body fat percentage than participants in the Diary and Control groups (e.g., BMI and body fat percentages reduced by 0.24 kg/m^2^ and 0.48%, respectively, more in the Activity Band PLUS group than in the Control group). Although these differences were in line with previous findings,^49^, ^50^, ^51^ they may not be clinically important. In an analysis of the Framingham Heart Study cohort, a 1-SD higher level of visceral adipose tissue was associated with a greater incidence of cardiovascular disease (hazard ratio=1.44, 95% CI=1.08,1.92).^52^ Given an average BMI of 26.8 (SD=5.3) kg/m^2^ in the study population, differences in BMI of 0.3 kg/m^2^ between groups represent 5.6% of an SD difference. Additional research is needed to determine whether such small changes in body composition are associated with decreased risk of morbidity and whether implementing the Activity Band PLUS intervention over a longer follow-up might lead to greater sustained reductions in body composition measures. In these analyses, it was also found that the Activity Band PLUS group had more positive attitudes toward PA self-monitoring than the Diary group and had stronger intentions to pay attention to how much PA they would do in the next 12 weeks than the Control group. These findings are in line with previous analyses demonstrating that higher levels of PA and exercise maintenance may be associated with more positive attitudes and intentions related to PA self-monitoring.^53^ This study adds to the current literature by suggesting that coupling activity trackers with virtual coaching may be associated with more positive attitudes and intentions related to PA self-monitoring.

The effectiveness of activity trackers coupled with virtual coaching,^54^, ^55^, ^56^ text messaging,^57^^,^^58^ personal coaching,^17^^,^^59^, ^60^, ^61^, ^62^, ^63^, ^64^ or lifestyle interventions^49^ and the effectiveness of Internet-based interventions and more traditional interventions (e.g., telephone counseling) delivered without the support of activity trackers have been examined previously.^65^, ^66^, ^67^ Although both activity and nonactivity tracker–based interventions have been shown to be effective,^66^^,^^68^^,^^69^ there is evidence that the former may be more effective.^66^ In a recent systematic review, activity tracker interventions facilitated greater increases in daily steps (standardized mean difference=0.24, 95% CI=0.16, 0.33), moderate- to vigorous-intensity PA (0.27, 95% CI=0.15, 0.39), and energy expenditure (0.28, 95% CI=0.03, 0.54) than nonactivity tracker–based interventions.^66^ This study adds to the literature by demonstrating that activity trackers paired with virtual coaching might be effective. In future studies, researchers may wish to compare the effectiveness of a variety of virtual coaching programs to identify which program would be the best to pair with activity trackers.

Strengths of this trial included high participant adherence and objective assessments of the coprimary and most of the secondary outcomes.

### Limitations

There were 6 key limitations. First, the study population was likely more health conscious than the general inactive working United Kingdom adult population. Larger effects might have been observed in less health conscious populations. Second, the activity trackers did not display data in real time. Greater effects might have been observed had participants been able to monitor PA in real time. Third, the active ingredient in the Activity Band PLUS intervention could not be identified because limited engagement data were collected. To understand causal pathways and to optimize intervention delivery, additional research is needed to determine what drove the observed effects. Fourth, although participants were instructed to fast for 1 hour before their baseline and follow-up visits, the half-life of nicotine and caffeine is 2–5 hours.^70^, ^71^, ^72^ It is possible that the participants allocated to the Activity Band PLUS group may have become more health conscious over the course of the trial than the participants randomized to the other groups and may have consequently modified their caffeine and nicotine intake. Given that the nicotine and caffeine use are associated with heart rate,^71^^,^^73^ a component of the cardiorespiratory estimation, bias may have arisen from differential nicotine/caffeine use in the intervention arms. Fifth, given that the measure of cardiorespiratory fitness was estimated on the basis of a heart rate response to only 1 submaximal ramped treadmill test at baseline and 1 at follow-up, the possibility of some measurement error from day-to-day variability in exercise heart rate response cannot be excluded entirely. Last, given that this study tested a large number of secondary outcomes, some of the observed associations may have arisen by chance.

## CONCLUSIONS

No overall difference among intervention groups was observed, but there was evidence that activity trackers coupled with virtual coaching may facilitate increases in PAEE and improve some secondary outcomes. More research is needed to more fully understand the pathways of impact.

## Appendix SUPPLEMENTAL MATERIAL

Image, application 1
