# Supplementary material for: Effectiveness of Minimal Contact Interventions: An RCT
Source: Am J Prev Med. 2021 Mar;60(3):e111–21. doi: 10.1016/j.amepre.2020.10.010 (PMC7899959; doi:10.1016/j.amepre.2020.10.010)
Supplement: Supplementary file 1 [file mmc1.pdf]

Appendix Figure 1. Study timeline.

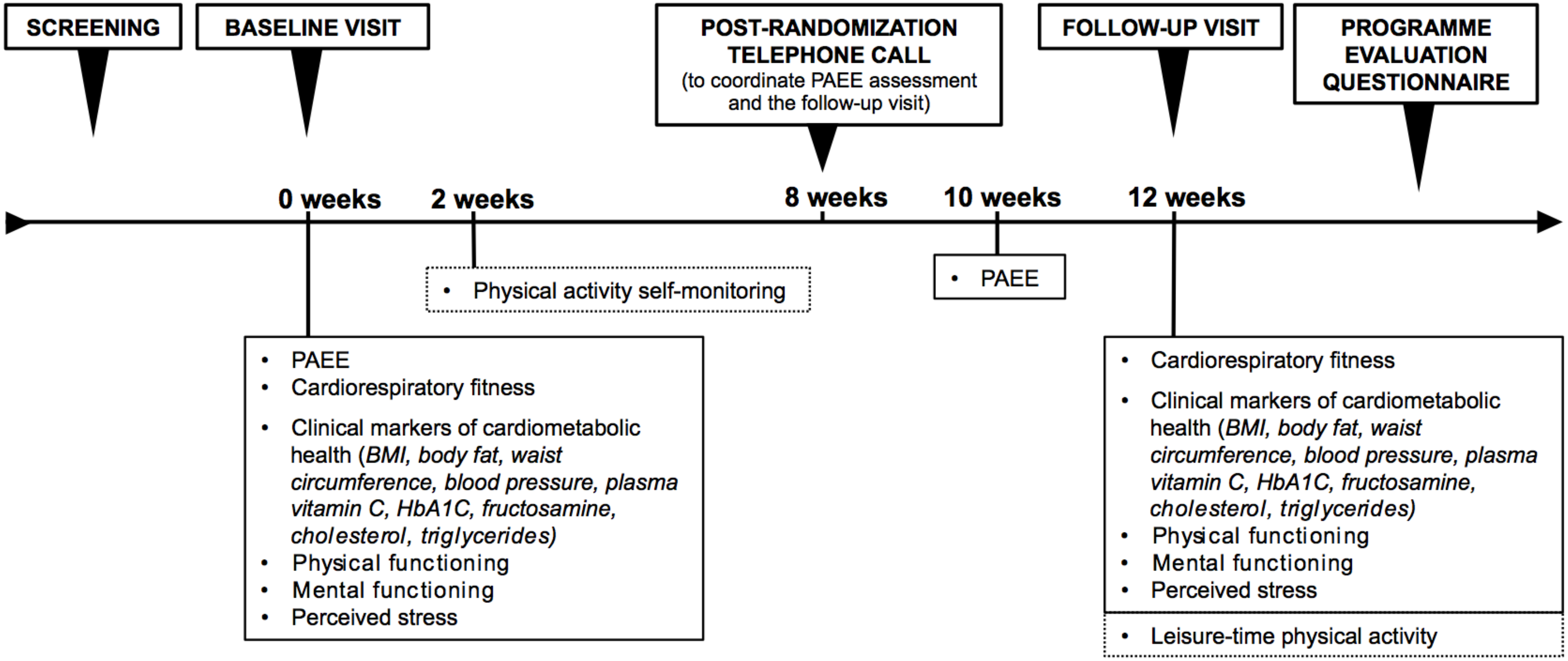

PAEE, physical activity energy expenditure.

Appendix Table 1. Items Included in the Calculation of Each of the Theory of Planned Behavior Constructs

Variable	Items	Response options
Becoming physically active		
Attitudes	1. Increasing how much physical activity I do over the next 12 weeks will be…	(1=Harmful; 7=Beneficial)
	2. Increasing how much physical activity I do over the next 12 weeks will be…	(1=Unpleasant; 7=Pleasant)
	3. Increasing how much physical activity I do over the next 12 weeks will be…	(1=Bad; 7=Good)
	4. Increasing how much physical activity I do over the next 12 weeks will be…	(1=Worthless; 7=Valuable)
	5. Increasing how much physical activity I do over the next 12 weeks will be…	(1=Unenjoyable; 7=Enjoyable)
Intentions	1. I intend to increase how much physical activity I do over the next 12 weeks.	(1=Strongly disagree; 7=Strongly agree)
	2. I will try to increase how much physical activity I do over the next 12 weeks.	(1=Strongly disagree; 7=Strongly agree)
Subjective norms	1. The people whose opinions I value would approve of me increasing how much physical activity I do over the next 12 weeks.	(1=Strongly disagree; 7=Strongly agree)
	2. It is expected of me that I increase how much physical activity I do over the next 12 weeks.	(1=Strongly disagree; 7=Strongly agree)
Perceived behavioral control	1. It is up to me whether or not I increase how much physical activity I do over the next 12 weeks.	(1=Strongly disagree; 7=Strongly agree)
	2. If I wanted to I could increase how much physical activity I do over the next 12 weeks.	(1=Strongly disagree; 7=Strongly agree)
Physical activity self-monitoring		

Attitudes	1.	Paying regular attention to how much physical activity I do over the next 12 weeks will be…	(1=Harmful; 7=Beneficial)
	2.	Paying regular attention to how much physical activity I do over the next 12 weeks will be…	(1=Unpleasant; 7=Pleasant)
	3.	Paying regular attention to how much physical activity I do over the next 12 weeks will be…	(1=Bad; 7=Good)
	4.	Paying regular attention to how much physical activity I do over the next 12 weeks will be…	(1=Worthless; 7=Valuable)
	5.	Paying regular attention to how much physical activity I do over the next 12 weeks will be…	(1=Unenjoyable; 7=Enjoyable)
Intentions	1.	I intend to pay regular attention to how much physical activity I do over the next 12 weeks.	(1=Strongly disagree; 7=Strongly agree)
	2.	I will try to pay regular attention to how much physical activity I do over the next 12 weeks.	(1=Strongly disagree; 7=Strongly agree)
Subjective norms	1.	The people whose opinions I value would approve of me paying regular attention to how much physical activity I do over the next 12 weeks.	(1=Strongly disagree; 7=Strongly agree)
	2.	It is expected of me that I pay regular attention to how much physical activity I do over the next 12 weeks.	(1=Strongly disagree; 7=Strongly agree)
Perceived behavioral control	1.	It is up to me whether or not I pay regular attention to how much physical activity I do each day over the next 12 weeks.	(1=Strongly disagree; 7=Strongly agree)
	2.	If I wanted to I could pay regular attention to how much physical activity I do over the next 12 weeks.	(1=Strongly disagree; 7=Strongly agree)

Appendix Table 2. Number of Participants With Follow-up Data

Variable	Intention-to-treat population				Per-protocol population			
	Control	Diary	Activity band	Activity band PLUS	Control	Diary	Activity band	Activity band PLUS
Physical activity energy expenditure, $kJ\cdot kg^{-1}\cdot day^{-1}$	108	112	106	106	108	94	63	68
Fitness, ml $O_2\cdot kg^{-1}\cdot min^{-1}$	108	113	110	109	108	97	66	70
BMI, kg/m^2	114	116	113	111	114	97	67	71
Body fat, %	114	117	113	111	114	98	67	71
Waist, cm	114	117	113	110	114	98	67	70
Systolic blood pressure, mmHg	114	117	113	111	114	98	67	71
Diastolic blood pressure, mmHg	114	117	113	111	114	98	67	71
Plasma vitamin C, µmol/l	113	115	111	108	113	96	66	68
HbA1c, mmol/mol	113	115	112	111	113	96	67	71
Fructosamine, micromol/l	113	115	110	109	113	96	66	69
Total cholesterol, mmol/l	114	115	112	111	114	96	67	71
HDL cholesterol, mmol/l	114	115	112	111	114	96	67	71
LDL cholesterol, mmol/l	114	115	112	110	114	96	67	70
Total/HDL cholesterol ratio, %	114	115	112	111	114	96	67	71
Triglycerides, mmol/l	114	115	112	111	114	96	67	71
Physical functioning	117	118	115	114	117	98	68	72
Mental functioning	117	118	115	114	117	98	68	72
Perceived Stress Scale (Range: 0–16)	117	118	115	114	117	98	68	72
Leisure-time physical activity, MET hours/day[a]	116	118	115	114	116	98	68	72
Physical activity self-monitoring (Range: 1–7)[b]								
Attitudes	113	109	108	111	113	91	66	70
Intentions	115	111	108	111	115	92	66	70
Subjective norms	115	111	109	111	115	92	67	70
Perceived behavioral control	115	111	109	109	115	92	67	69

[a]Leisure-time physical activity: assessed at 12 weeks follow-up.
[b]Physical activity self-monitoring: assessed at 2 weeks post randomization.

HDL, high-density lipoprotein; LDL, low-density lipoprotein.

Appendix Figure 2. Baseline and follow-up levels of physical activity energy expenditure (PAEE) and cardiorespiratory fitness and corresponding changes over the 12 week follow-up period.

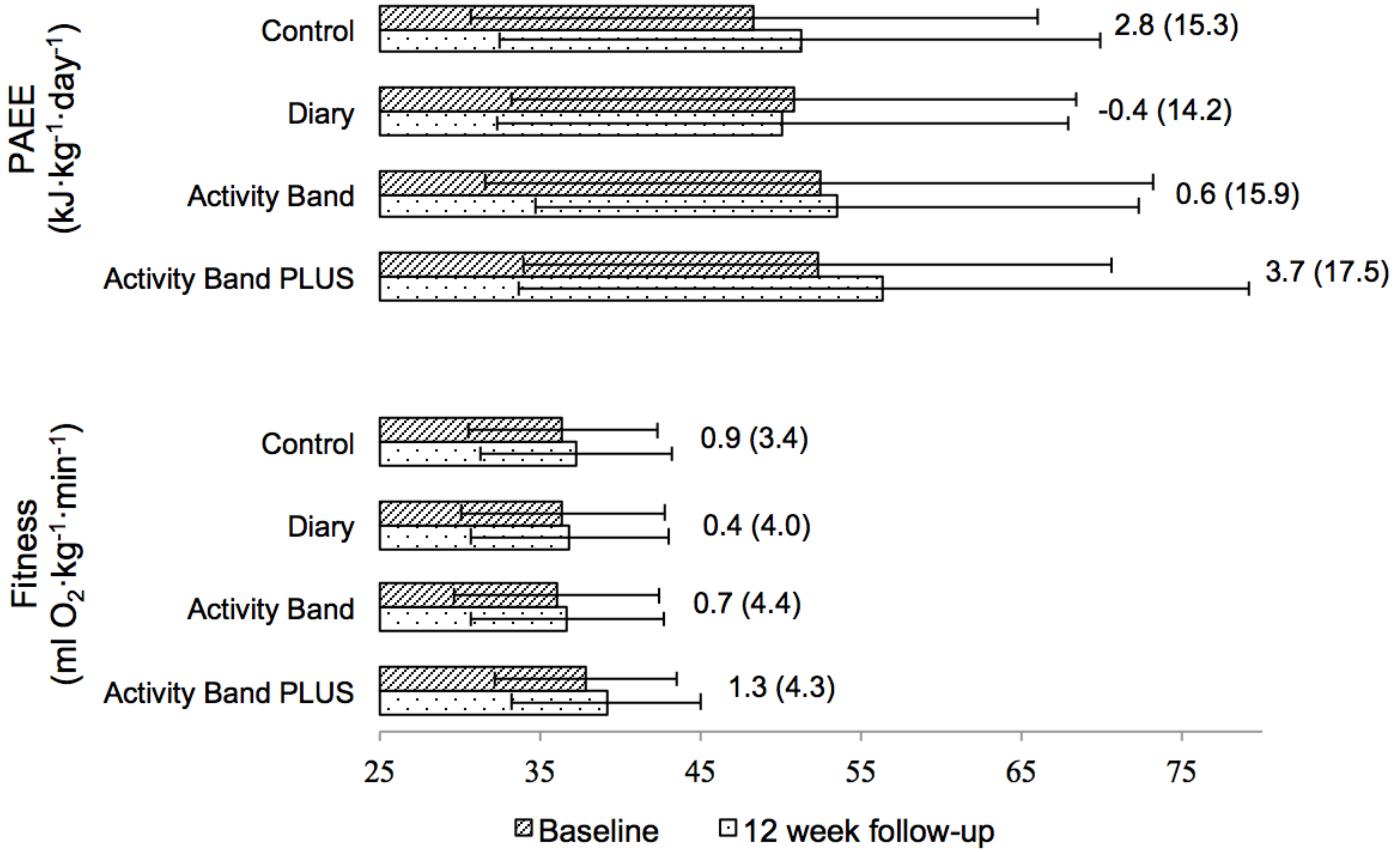

Note: Values represent means (SDs) of change from baseline to 12 week follow-up. The sample sizes upon which the values are based correspond to the sample sizes for each randomized group reported in Figure 1 (PAEE: Control=121, Diary=124, Activity Band=122, Activity Band PLUS=121; Fitness: Control=108, Diary=112, Activity Band=106, Activity Band PLUS=106).

Appendix Table 3. Baseline-Adjusted Differences in Mean Changes in Physical Activity Expenditure (PAEE) and Cardiorespiratory Fitness Between Randomized Groups With and Without Adjustment for the Randomization Stratifiers - Age, Sex, and BMI (95% CIs)

Variable	Without adjustment for the randomization stratifiers	With adjustment for the randomization stratifiers
PAEE, $kJ \cdot kg^{-1} \cdot day^{-1}$		
Diary vs control	–3.22 (–7.01, 0.58)	–3.19 (–6.96, 0.58)
Activity band vs control	–2.14 (–5.96, 1.68)	–2.13 (–5.92, 1.66)
Activity band PLUS vs control	0.99 (–2.83, 4.82)	1.05 (–2.75, 4.85)
Activity band vs diary	1.07 (–2.71, 4.86)	1.07 (–2.69, 4.82)
Activity band PLUS vs diary	4.21 (0.42, 8.00)	4.24 (0.48, 8.01)
Activity band PLUS vs activity band	3.13 (–0.68, 6.95)	3.17 (–0.61, 6.96)
Fitness, ml $O_2 \cdot kg^{-1} \cdot min^{-1}$		
Diary vs control	–0.43 (–1.46, 0.59)	–0.43 (–1.46, 0.59)
Activity band vs control	–0.12 (–1.16, 0.92)	–0.12 (–1.16, 0.92)
Activity band PLUS vs control	0.45 (–0.60, 1.49)	0.45 (–0.60, 1.49)
Activity band vs diary	0.31 (–0.72, 1.35)	0.31 (–0.72, 1.35)
Activity band PLUS vs diary	0.88 (–0.16, 1.91)	0.88 (–0.15, 1.91)
Activity band PLUS vs activity band	0.57 (–0.48, 1.61)	0.57 (–0.48, 1.61)

Appendix Table 4. Intervention Effects, Per-Protocol Population (Mean Differences, 95% CIs)

Variable	Diary vs control	Activity band vs control	Activity band PLUS vs control	Activity band vs diary	Activity band PLUS vs diary	Activity band PLUS vs activity band
Differences in mean changes between groups in the secondary outcomes that were assessed at baseline and at 12-weeks follow-up						
PAEE, $kJ\cdot kg^{-1}\cdot day^{-1}$[a]	–3.42 (–7.24, 0.41)	–3.48 (–7.74, 0.78)	–0.65 (–4.83, 3.54)	–0.07 (–4.46, 4.33)	2.77 (–1.55, 7.09)	2.84 (–1.87, 7.54)
PAEE, $kJ\cdot kg^{-1}\cdot day^{-1}$[b]	–3.24 (–7.07, 0.59)	–3.49 (–7.75, 0.77)	–0.79 (–4.98, 3.40)	–0.25 (–4.64, 4.15)	2.45 (–1.88, 6.78)	2.70 (–2.02, 7.41)
Fitness, ml $O_2\cdot kg^{-1}\cdot min^{-1}$	–0.10 (–1.17, 0.96)	0.14 (–1.06, 1.33)	0.38 (–0.79, 1.55)	0.24 (–0.99, 1.47)	0.48 (–0.72, 1.69)	0.25 (–1.07, 1.57)
BMI, kg/m^2	0.06 (–0.17, 0.28)	–0.14 (–0.39, 0.11)	–0.36 (–0.61, –0.12)	–0.20 (–0.46, 0.06)	–0.42 (–0.67, –0.16)	–0.22 (–0.50, 0.06)
Body fat, %	0.08 (–0.35, 0.51)	–0.22 (–0.69, 0.26)	–0.72 (–1.19, –0.25)	–0.30 (–0.79, 0.20)	–0.80 (–1.28, –0.31)	–0.50 (–1.03, 0.03)
Waist, cm	–0.43 (–4.13, 3.27)	–5.18 (–9.30, –1.06)	–1.02 (–5.08, 3.04)	–4.75 (–9.02, –0.48)	–0.59 (–4.80, 3.62)	4.16 (–0.42, 8.74)
Systolic blood pressure, mmHg	1.07 (–1.27, 3.41)	–0.30 (–2.90, 2.31)	–0.49 (–3.05, 2.07)	–1.37 (–4.07, 1.33)	–1.56 (–4.21, 1.10)	–0.19 (–3.08, 2.70)
Diastolic blood pressure, mmHg	0.64 (–0.91, 2.19)	–0.32 (–2.04, 1.41)	–1.01 (–2.71, 0.68)	–0.96 (–2.74, 0.83)	–1.65 (–3.41, 0.10)	–0.70 (–2.61, 1.22)
Plasma vitamin C, µmol/l	1.79 (–3.70, 7.28)	–1.51 (–7.57, 4.55)	–1.90 (–7.91, 4.11)	–3.30 (–9.61, 3.00)	–3.69 (–9.95, 2.56)	–0.39 (–7.15, 6.38)
HbA1c, mmol/mol	–0.13 (–0.66, 0.40)	0.10 (–0.48, 0.69)	0.21 (–0.37, 0.79)	0.23 (–0.38, 0.84)	0.34 (–0.26, 0.94)	0.11 (–0.54, 0.76)
Fructosamine, micromol/l	4.28 (–2.70, 11.26)	–1.38 (–9.15, 6.39)	–0.06 (–7.67, 7.55)	–5.66 (–13.73, 2.41)	–4.34 (–12.25, 3.58)	1.32 (–7.30, 9.94)
Total cholesterol, mmol/l	–0.01 (–0.16, 0.15)	0.03 (–0.14, 0.20)	–0.11 (–0.27, 0.06)	0.03 (–0.14, 0.21)	–0.10 (–0.28, 0.07)	–0.13 (–0.32, 0.06)
HDL cholesterol, mmol/l	0.02 (–0.04, 0.08)	0.01 (–0.05, 0.08)	0.02 (–0.05, 0.08)	–0.01 (–0.08, 0.06)	–0.01 (–0.07, 0.06)	0.002 (–0.07, 0.08)
LDL cholesterol, mmol/l	–0.01	–0.01	–0.07	0.0005	–0.06	–0.06

	(–0.14, 0.12)	(–0.16, 0.13)	(–0.21, 0.07)	(–0.15, 0.15)	(–0.20, 0.09)	(–0.22, 0.10)
Total/HDL cholesterol ratio, %	–0.09	–0.04	–0.11	0.05	–0.02	–0.07
	(–0.23, 0.05)	(–0.20, 0.12)	(–0.26, 0.05)	(–0.11, 0.21)	(–0.18, 0.14)	(–0.24, 0.11)
Triglycerides[c]	1.01	0.99	0.88	0.99	0.87	0.88
	(0.90, 1.13)	(0.88, 1.13)	(0.77, 0.99)	(0.87, 1.13)	(0.77, 0.99)	(0.77, 1.01)
Physical functioning	–1.58	–0.62	–0.42	0.96	1.16	0.20
	(–3.49, 0.33)	(–2.74, 1.50)	(–2.50, 1.67)	(–1.24, 3.16)	(–1.00, 3.33)	(–2.15, 2.55)
Mental functioning	–0.18	–0.66	1.37	–0.48	1.55	2.03
	(–2.35, 2.00)	(–3.07, 1.76)	(–1.00, 3.75)	(–2.99, 2.03)	(–0.92, 4.01)	(–0.65, 4.71)
Perceived Stress Scale (Range: 0–16)	0.06	–0.22	–0.02	–0.29	–0.09	0.20
	(–0.63, 0.76)	(–1.00, 0.55)	(–0.78, 0.74)	(–1.09, 0.51)	(–0.87, 0.70)	(–0.65, 1.06)
Mean differences between groups in secondary outcomes that were assessed only during follow-up						
Leisure-time physical activity, MET hours/day[d]	–1.37	–0.79	–0.49	0.57	0.88	0.31
	(–2.57, –0.17)	(–2.13, 0.54)	(–1.80, 0.82)	(–0.80, 1.95)	(–0.48, 2.24)	(–1.17, 1.78)
Physical activity self-monitoring (Range: 1–7)						
Attitudes	–0.06	0.24	0.20	0.31	0.26	–0.05
	(–0.33, 0.21)	(–0.05, 0.54)	(–0.09, 0.49)	(–0.003, 0.61)	(–0.05, 0.56)	(–0.37, 0.28)
Intentions	0.41	0.58	0.62	0.17	0.21	0.04
	(0.04, 0.77)	(0.18, 0.98)	(0.23, 1.02)	(–0.25, 0.59)	(–0.20, 0.63)	(–0.41, 0.49)
Subjective norms	0.57	0.30	0.31	–0.27	–0.27	0.002
	(0.18, 0.96)	(–0.13, 0.73)	(–0.12, 0.73)	(–0.72, 0.18)	(–0.71, 0.18)	(–0.48, 0.48)
Perceived behavioral control	–0.10	0.01	0.09	0.10	0.18	0.08
	(–0.38, 0.19)	(–0.31, 0.32)	(–0.22, 0.40)	(–0.22, 0.43)	(–0.14, 0.51)	(–0.27, 0.43)

[a]Original per-protocol analysis (n, Control=121, Diary=98, Activity Band=69, Activity Band PLUS=73).
[b]Follow-up values for participants only included if they accrued ≥48 hours of activity-heart rate data with ≥8 hours of data in each quadrant of the day.
[c]Intervention effects for triglycerides are presented as ratios of geometric means, and therefore 1 is the value corresponding to no effect.
[d]Activities assessed as part of leisure-time physical activity: swimming, backpacking, walking for pleasure, cycling, gardening, do-it-yourself activities, fitness/exercising activities, dancing, running, bowling, tennis/badminton, squash, table tennis, golf, football/rugby/hockey, cricket, rowing, netball/volleyball/basketball, fishing, horse riding, snooker/billiards/darts, musical instruments playing/singing, ice skating, sailing/windsurfing/boating, and martial arts/boxing/wrestling.

PAEE, physical activity energy expenditure; HDL, high-density lipoprotein; LDL, low-density lipoprotein.

Appendix Table 5. Summary of the Process Measures Evaluated at Follow-up[a,b]

Variable	Control	Diary	Activity band	Activity band PLUS
Days off work due to illness, mean (SD)	0.8 (1.8)	1.8 (6.5)	2.8 (7.5)	1.5 (6.0)
Job satisfaction (Range: 1–7), mean (SD)	5.3 (1.3)	5.1 (1.4)	5.1 (1.4)	5.2 (1.4)
Contact with other study participants, % (n)	47.0 (55)	39.8 (47)	56.5 (65)	50.0 (57)
Physical activity tracking, % (n)	11.1 (13)	83.1 (98)	47.8 (55)	70.2 (80)
Self-weighing, >once/month, % (n)	53.0 (62)	44.9 (53)	45.2 (52)	74.6 (85)
Use of physical activity advice websites, % (n)	17.9 (21)	22.9 (27)	22.6 (26)	51.8 (59)

[a]Number of participants: Days off work due to illness (Control=116, Diary=117, Activity Band=111, Activity Band PLUS=114); Job satisfaction/contact with other study participants/self-monitoring behaviors/use of physical activity advice websites (Control=117, Diary=118, Activity Band=115, Activity Band PLUS=114).

[b]Days off work due to illness: *In the past 12-weeks, how many days have you been off work due to ill health? (do not include days off for any other reason)*; Job satisfaction: *How satisfied are you with your job as a whole?* Responses were on a 7-point scale ranging from *Very unsatisfied* to *Very satisfied*. Contact with other study participants: *During the study, to what extent did you share your experiences with another person/other people who were also taking part in the study? A lot*, *Quite a lot*, or *A little*=had contact with other study participants; *Not at all*=did not have contact with other study participants. Physical activity tracking: *Thinking back over the last 4 weeks, in a typical week to what extent did you keep track of your physical activity, for example by using a diary, chart or pedometer? A little*, *Quite a lot*, or *A lot*=tracked physical activity levels; *Not at all*=did not track physical activity levels. Self-weighing: *During the past 12 weeks, how often have you weighed yourself? About once a month or less* or *Never*=self-weighing ≤once/month; *Every day*, *Most days*, *2-3 times/week* or *About once/week or less*=self-weighing >once/month. Use of physical activity advice websites: *During the past 4 weeks, how often did you visit websites which give advice about increasing physical activity? Every day*, *Twice/week*, *About once/week or less*, or *About once a month or less*=used physical activity advice websites; *Never*=not did not use physical activity advice websites.

Appendix Table 6. Percentages of Participants (n) Assessed in Each Season at the Baseline and Follow-up Assessments

Participants	Control	Diary	Activity band	Activity band PLUS
Baseline, n	121	124	122	121
Autumn	13.2 (16)	18.5 (23)	17.2 (21)	13.2 (16)
Winter	31.4 (38)	28.2 (35)	30.3 (37)	33.1 (40)
Spring	28.9 (35)	29.8 (37)	28.7 (35)	30.6 (37)
Summer	26.4 (32)	23.4 (29)	23.8 (29)	23.1 (28)
Follow-up, n	109	113	110	109
Autumn	22.9 (25)	24.8 (28)	23.6 (26)	21.1 (23)
Winter	14.7 (16)	15.0 (17)	12.7 (14)	14.7 (16)
Spring	27.5 (30)	28.3 (32)	30.0 (33)	32.1 (35)
Summer	34.9 (38)	31.9 (36)	33.6 (37)	32.1 (35)

Note: Seasons were based on the dates of the first day of activity monitoring: Autumn (September–November); Winter (December–February); Spring (March–May); Summer (June–August).
